# Supplementary material for: Protocol for the development of SPIRIT and CONSORT extensions for randomised controlled trials with surrogate primary endpoints: SPIRIT-SURROGATE and CONSORT-SURROGATE
Source: BMJ Open. 2022 Oct 11;12(10):e064304. doi: 10.1136/bmjopen-2022-064304 (PMC9557267; doi:10.1136/bmjopen-2022-064304)
Supplement: Supplementary data [file bmjopen-2022-064304supp001.pdf]

Supplementary File

Supplementary Table 1: Delphi survey stakeholder categories (approximate target sample sizes, and identification strategies)

Stakeholder category	Approx. sample: % (n)	Identification strategies
**Methodologists, Statisticians, Healthcare professionals, and Epidemiologists based in academia and healthcare industry**	~50% (100)	• Professional bodies and networks such as the UKCRC CTU network<br>• Conferences and meetings such as the 6th International Clinical Trials Methodology Conference 2022 (ictmc.org/)<br>• Corresponding authors of protocols and trials identified in the Targeted Review (Phase 1)<br>• Projects team members professional contacts
**Surrogate content experts**	~20% (40)	• Corresponding authors of literature included in the scoping review (Phase 1)<br>• Project team members professional contacts
**Public and patient representatives**	~10% (20)	• Patient and public engagement forums such as European Patient Forum (www.eu-patient.eu)<br>• Consumers/patient and public involvement experts in the MRC-NIHR Trials Methodology Research Partnership<br>• Project team members professional contacts
**Funders, regulators, HTA experts and clinical guideline developers**	~10% (20)	• Funding committees' members (with special interest in clinical trials) such as the NIHR/MRC<br>• Health technology assessment bodies such as NICE<br>• Regulatory bodies such as MHRA, FDA<br>• Project team members professional contacts
**Journal editors**	~10% (20)	• Editorial groups such as the International Committee of Medical Journal Editors<br>• Project team professional contacts
**A call will be placed on the project website and social media pages and targeted invitations for potential participants from any stakeholder category to register**<br>***Weighting will not be done during analysis**		
