# Supplementary material for: Protocol for the development of SPIRIT and CONSORT extensions for randomised controlled trials with surrogate primary endpoints: SPIRIT-SURROGATE and CONSORT-SURROGATE
Source: BMJ Open. 2022 Oct 11;12(10):e064304. doi: 10.1136/bmjopen-2022-064304 (PMC9557267; doi:10.1136/bmjopen-2022-064304)
Supplement: Author's manuscript [file bmjopen-2022-064304.draft_revisions.pdf]

BMJ Open is committed to open peer review. As part of this commitment we make the peer review history of every article we publish publicly available.

When an article is published we post the peer reviewers' comments and the authors' responses online. We also post the versions of the paper that were used during peer review. These are the versions that the peer review comments apply to.

The versions of the paper that follow are the versions that were submitted during the peer review process. They are not the versions of record or the final published versions. They should not be cited or distributed as the published version of this manuscript.

BMJ Open is an open access journal and the full, final, typeset and author-corrected version of record of the manuscript is available on our site with no access controls, subscription charges or pay-per-view fees (http://bmjopen.bmj.com).

If you have any questions on BMJ Open's open peer review process please email
info.bmjopen@bmj.com

BMJ Open

# Protocol for the development of SPIRIT and CONSORT extensions for randomised controlled trials with surrogate primary endpoints: SPIRIT-SURROGATE and CONSORT-SURROGATE.



| | |
|---|---|
| Journal: | *BMJ Open* |
| Manuscript ID | bmjopen-2022-064304 |
| Article Type: | Protocol |
| Date Submitted by the Author: | 28-Apr-2022 |





| | |
|---|---|
| Complete List of Authors: | Manyara, Anthony; University of Glasgow<br>Davies, Philippa ; University of Bristol<br>Stewart, Derek; Patient and Public Involvement lead<br>Weir, Christopher; University of Edinburgh, Edinburgh Clinical Trials Unit, Usher Institute<br>Young, Amber; University Hospitals Bristol NHS Foundation Trust, Paediatric Anaesthesia; University of Bristol, Canynge Hall<br>Butcher, Nancy; Sick Kids, Child Health Evaluative Sciences<br>Bujkiewicz, Sylwia; Biostatistics Research Group, Department of Health Sciences, University of Leicester<br>Chan, An-Wen; University of Toronto, Medicine<br>Collins, Gary; Oxford University, Centre for Statistics in Medicine<br>Dawoud, Dalia; National Institute for Health and Care Excellence<br>Offringa, Martin; The Hospital for Sick Children, Child Health Evaluation Sciences<br>Ouwens, Mario; AstraZeneca<br>Ross, Joseph; Yale University School of Medicine, Internal Medicine<br>Taylor, Rod; University of Glasgow<br>Ciani, Oriana ; Università Bocconi |





| | |
|---|---|
| Keywords: | STATISTICS & RESEARCH METHODS, Protocols & guidelines < HEALTH SERVICES ADMINISTRATION & MANAGEMENT, GENERAL MEDICINE (see Internal Medicine), PUBLIC HEALTH |
| | |



SCHOLARONE™
Manuscripts

# Protocol for the development of SPIRIT and CONSORT extensions for randomised controlled trials with surrogate primary endpoints: SPIRIT-SURROGATE and CONSORT-SURROGATE.

Anthony Muchai Manyara[1], Philippa Davies[2], Derek Stewart[3], Christopher J Weir[4], Amber E Young[2], Nancy J. Butcher[5,6], Sylwia Bujkiewicz[7]. An-Wen Chan[8,9], Gary S Collins[10], Dalia Dawoud[11], Martin Offringa[5], Mario Ouwens[12], Joseph S. Ross[13,14], Rod S Taylor[1,15], Oriana Ciani[16]

1 MRC/CSO Social and Public Health Sciences Unit, Institute of Health and Wellbeing, University of Glasgow, Glasgow, UK

2 Population Health Sciences, Bristol Medical School, University of Bristol, Bristol, UK

3 Patient and Public Involvement lead, UK

4 Edinburgh Clinical Trials Unit, Usher Institute, University of Edinburgh, Edinburgh, UK

5 Child Health Evaluative Sciences, The Hospital for Sick Children Research Institute, Toronto, Canada

6 Department of Psychiatry, University of Toronto, Toronto, Canada

7 Biostatistics Research Group, Department of Health Sciences, University of Leicester, Leicester, UK

8 Women's College Institute Research Institute, Toronto, Canada

9 Department of Medicine, University of Toronto, Toronto, Canada

10 Centre for Statistics in Medicine, Nuffield Department of Orthopaedics, Rheumatology & Musculoskeletal Sciences, University of Oxford, Oxford, UK

11 Science, Evidence and Analytics Directorate, Science Policy and Research Programme, National Institute for Health and Care Excellence, London, UK

12 AstraZeneca, Weesp, Netherlands

13 Department of Health Policy and Management, Yale School of Public Health, New Haven, CT, USA



For peer review only - http://bmjopen.bmj.com/site/about/guidelines.xhtml

14 Section of General Medicine, Department of Internal Medicine, Yale School of Medicine, New Haven, CT, USA

15 Robertson Centre for Biostatistics, Institute of Health and Well Being, University of Glasgow, Glasgow, UK

16 SDA Bocconi School of Management, Milan, Italy

Correspondence to Anthony Manyara; Anthony.Manyara@glasgow.ac.uk

MRC/CSO Social and Public Health Sciences Unit, Institute of Health and Wellbeing, University of Glasgow, Glasgow, UK

For peer review only



For peer review only - http://bmjopen.bmj.com/site/about/guidelines.xhtml

# Abstract

*Introduction*

Randomised controlled trials (RCTs) may use surrogate endpoints as substitutes and predictors of patient/participant-relevant final outcomes (e.g., survival, health related quality of life). Translation of effects measured on a surrogate endpoint into health benefits for patients/participants is dependent on the validity of the surrogate, hence more accurate and transparent reporting on surrogate endpoints is needed to limit misleading interpretation of trial findings. However, there is currently no explicit guidance for the reporting of such trials. Therefore, we aim to develop extensions to the SPIRIT (Standard Protocol Items: Recommendations for Interventional Trials) and CONSORT (Consolidated Standards of Reporting Trials) reporting guidelines to improve the design and completeness of reporting of RCTs and their protocols using a surrogate endpoint as a primary outcome.

*Methods and analysis*

The project will have four phases: Phase 1 (literature reviews) to identify candidate reporting items to be rated in a Delphi study; Phase 2 (Delphi study) to rate the importance of items identified in Phase 1 and receive suggestions for additional items; Phase 3 (consensus meeting) to agree on final set of items for inclusion in the extensions; and Phase 4 (knowledge translation) to engage stakeholders and disseminate the project outputs through various strategies including peer-reviewed publications. Patient and public involvement will be embedded into all project phases.

*Ethics and dissemination*

Ethical approval will be sought from University of Glasgow Ethics Committee. The findings will be published in open-access peer-reviewed publications and presented in conferences, meetings, and relevant forums.


For peer review only



For peer review only - http://bmjopen.bmj.com/site/about/guidelines.xhtml

## Strengths and limitations of this study

- We will follow the EQUATOR Network's recommended steps for developing a health research reporting guideline.
- The Delphi study will target an international and multidisciplinary group of participants.
- Patient and public involvement will be integrated in all phases of the study.
- Although we will target an international participation, Delphi study and consensus meeting will be conducted in English which could limit participation from non-English speaking settings.

For peer review only



For peer review only - http://bmjopen.bmj.com/site/about/guidelines.xhtml

# Introduction

Evidence for the effectiveness of interventions should ideally come from randomised controlled trials (RCTs) [1, 2] that assess a patient /study participant relevant final outcome (PRFO): a measurement that reflects how an individual feels, functions, or survives [3] such as mortality or health-related quality of life. Nevertheless, in order to meet the scientific, ethical, and regulatory requirements, the conduct and delivery of many RCTs has become increasingly resource and time-intensive [4]. By reducing follow up time, sample size, and cost, a surrogate endpoint that substitutes for a PRFO, can provide a potentially attractive solution for improving trial efficiency [5].

Over the last decade, a number of biomarkers [an objectively measured molecular, histologic, radiographic, or physiologic characteristic used as an indicator of response to an intervention[5]] have been accepted as surrogate endpoints in the regulatory approval of pharmaceuticals and medical devices e.g., blood pressure and low density lipoprotein-cholesterol within the cardiovascular disease context [6]. However, for a wider range of interventions including surgical, organizational, and public health interventions, the so-called intermediate outcomes [i.e., an outcome that can be measured earlier than an effect on PFRO and is predictive of the intervention effect on the PFRO] [3]] have been used with the aim of replacing and predicting for a PRFO (e.g., hospice enrolment instead of mortality with an intervention aimed at improving end of life care [7]; fruit and vegetable consumption instead of cardiovascular events for a behavioural intervention designed to improve cardiovascular risk [8]).

Despite their potential benefits, the use of surrogate endpoints in health and policy decision making remains controversial. Some regulatory approvals based on a surrogate endpoint have led to the use of interventions that have resulted in more harm than health benefit for patients due to the surrogate not being in the pathway of the PRFO [9, 10]. Additionally, RCTs using a surrogate endpoint primary outcome have been shown to overestimate treatment effects (adjusted odds ratios: 1.46, 95% CI: 1.05 to 2.04) compared with RCTs with PRFO as a primary outcome [11]. It is therefore important that RCTs using surrogate endpoints are appropriately designed and reported e.g., explicitly state that the primary outcome is a surrogate endpoint, give a clear rationale for its use, and provide evidence of its validation (i.e., prior evidence of the association between the intervention effect on the putative surrogate endpoint and PRFO) [12] However, a retrospective analysis of 626 RCTs published



For peer review only - http://bmjopen.bmj.com/site/about/guidelines.xhtml

in 2005 and 2006 found that 107 (17%) used a surrogate primary endpoint and of these, only a third discussed whether the surrogate endpoint was a valid predictor of health benefit on a PRFO [13]. Similarly, a more recent review of 220 cardiovascular intervention trials using surrogate biomarkers found that only 59 (27%) had confirmatory evidence validating the benefits of interventions on a PRFO [14].

Reporting guidelines can improve transparency and completeness in the reporting of RCTs at both the protocol and final report stages. The two prominent trial guidelines are: SPIRIT (Standard Protocol Items: Recommendations for Interventional Trials) 2013 statement: a 33-item checklist used to report RCT protocols [15]; and CONSORT (Consolidated Standards of Reporting Trials) 2010 statement is a 25-item checklist, which has improved reporting of completed trials [16, 17]. Although SPIRIT and CONSORT and related extensions, including the (SPIRIT-PRO [18], CONSORT-PRO [19]) and the ongoing CONSORT-Outcomes [20], provide general guidance on trial outcome reporting, there remains no standard evidence-based guideline for the reporting of RCTs with a surrogate primary outcome. Therefore, in this study, we aim to develop specific extensions to report RCT protocols and final trial reports that use a surrogate primary endpoint: SPIRIT-SURROGATE and CONSORT-SURROGATE, respectively. This protocol describes the methods that will be used in developing these extensions.

## Methods

Our methodology will be guided by: the EQUATOR (Enhancing the QUAlity and Transparency Of health Research) Network's recommended steps for developing a health research reporting guideline [21]; and methodological considerations used to develop other recent SPIRIT/CONSORT extensions (e.g., ACE [Adaptive designs CONSORT Extension] [22], CONSORT-ROUTINE [23]).

The project will be overseen by a Project Management Group (PMG) and an advisory Executive Committee (EC). The PMG includes the lead investigators (OC, RST) and project manager (AMM) responsible for the day-to-day management of the project plus project co-investigators (PD, AEY, CJW), and patient and public involvement lead (DS). The EC is an international and multidisciplinary group (JSR [chair], NJB, SB, AWC, GSC, DD, MO, MO) providing strategic oversight of the project and will contribute to the dissemination, endorsement, and implementation of developed extensions.



For peer review only - http://bmjopen.bmj.com/site/about/guidelines.xhtml

Figure 1 shows the project phases, timelines, activities in each phase, and the integrated knowledge translation and patient and public involvement which are described in more detail below.

## *Phase 1: Literature reviews*

A detailed protocol for this phase has been prepared, prospectively registered [25], and submitted for publication. Briefly, we will undertake two separate literature reviews that include a scoping review and a 'targeted review'. The scoping review, conducted using the Arksey and O'Malley six-stage methodological framework [26], will seek to explore *the current understanding, limitations, acceptability, and guidance on using surrogate endpoints in RCTs* and generate two outputs: candidate items ("long-list") for rating through a Delphi exercise; and a contact list of surrogate content experts who will be invited to participate in the exercise. Further, one of the issues to be explored in the scoping review will be an appropriate and comprehensive working definition of surrogate endpoint, starting from the most commonly reported definition in 2001 by the National Institutes of Health (i.e., a biomarker or laboratory measure intended to substitute and predict for a variable that reflects how patients feel, function or how long they survive) [5]. The targeted review will seek to systematically identify recent protocols and full reports of RCTs that have used surrogate endpoints as primary outcomes. It will serve two purposes: 1) identify researchers who have used surrogate endpoints who will be invited to participate in the Delphi exercise; and 2) the identified protocols and reports will inform a detailed contemporary analysis of the completeness of reporting of RCTs with surrogate endpoint primary outcomes.

## *Phase 2: Delphi study*

The primary objective of this phase will be to rate candidate items generated in Phase 1. Secondary objectives will be to identify additional items not included in the initial list; and allow for modifications in the wording of important items [23].

### *Study design and setting*

The Delphi methodology is a widely used consensus-building technique whose main features are: use of experts as participants, anonymity between participants, iterations and controlled feedback (to allow for "communication" and consensus building between participants), and summary of participant views [27-30]. Its key practical advantage is the non-requirement of face-to-face contact [30], enabling more participants and broader representation in terms of geography and key groups in the development of health research reporting guidelines [21].



For peer review only - http://bmjopen.bmj.com/site/about/guidelines.xhtml

Our Delphi study will be conducted online and facilitated by DelphiManager software (version 5.0), a bespoke software developed and maintained by the COMET (Core Outcome Measures in Effectiveness Trials) initiative (www.comet-initiative.org/), which has been used to develop other SPIRIT and CONSORT extensions [31, 32].

It is recommended that the number of Delphi study rounds is determined *a priori* [27]. To meet project timelines and reduce potential participant burden/fatigue, we propose two rounds will be conducted. Two Delphi rounds have been used to develop SPIRIT/CONSORT extensions for reporting: pilot RCTs [33]; RCTs using adaptive design [22]; interventions involving artificial intelligence [31, 34]; and social and psychological interventions [35]. However, if we fail to obtain consensus in most items after round 2 and based on advice from the EC, we will consider a third round [20, 23, 36].

*Sample size, recruitment, and inclusion criteria*

Delphi studies do not require formal sample size calculations, although the number and characteristics of participants need to be carefully considered [27, 29, 30, 37]. Consistent with the EQUATOR Network's guidance [21], we will seek to recruit an international multidisciplinary group of stakeholders, including trialists, methodologists, healthcare professionals, researchers, content experts, journal editors, patient and public representatives, funders, regulators, Health Technology Assessment experts and clinical guideline developers. Supplementary File 1 shows stakeholder categories, approximate target sample sizes, and strategies for access. We will target to have at least 200 participants register interest to take part in the study. A sample size of ≥200 will allow recruitment of at least 20 participants per stakeholder group and reasonable numbers (>70 participants) to complete the study, assuming ≥60% response rate of eligible participants and ≤40% attrition (in the context of completing all Delphi rounds), both as observed in previous Delphi studies that have developed extension guidelines [22, 23, 33, 34].

We will use purposive and snowball sampling (nonprobability sampling) to include participants [38]. Identification strategies will include: 1) professional contacts known to the PMG and EC; 2) relevant professional bodies and networks; 3) relevant conferences and meetings; 4) authors of records included in the scoping and targeted reviews (Phase 1); 5) a call for participants on project website and social media pages; and 6) asking registered participants to share link with other people, networks or organisations that would be interested in participating (see Supplementary table in Supplementary File). We acknowledge



For peer review only - http://bmjopen.bmj.com/site/about/guidelines.xhtml

that our target sample size may be challenging, particularly for some stakeholder categories (e.g., journal editors (n=20)); however, efforts will be made to reach as many of these stakeholders as possible.

Participant inclusion criteria will be: 1) expertise in surrogate endpoints (through authored literature) or self-reported interest and basic understanding of the concept of surrogacy; 2) registered interest, in English (nevertheless participation will be international), to participate during the allocated period. We will have no exclusion criteria. During registration, the following descriptive data on participants will be collected: self-identified stakeholder group (primary/secondary roles), clinical or research area (if actively involved), country of work, and self-reported basic understanding of surrogate endpoints.

*Data collection, analysis, and consensus definition*

Prior to the launch of the first survey round, a pilot (with n=15 participants) will be conducted to gauge user-friendliness, improve wording/logical flow, and identify any practical concerns [39]. Following the pilot, all registered participants will be emailed a web link prompting them to complete any of the Delphi rounds. The weblink will access the Delphi survey landing page that includes: a short text section emphasising the importance of completing the exercise [43], a participant information sheet, and a consent checkbox. Each round will be open for approximately four weeks, and the second round sent out 3-4 weeks after the closure of the first. Email reminders to complete the survey will be sent to participants to improve response rates. Participants who complete all Delphi rounds may opt to enter a prize draw to win one of two £100 vouchers [40].

Candidate Delphi items will be ranked using a Likert scale. There is no consensus on the ideal rating scale in Delphi studies [41]. We will use the Grading of Recommendations Assessment, Development and Evaluations (GRADE) 9-point scale [42], that has been used in other extensions [20, 22, 31-33, 40], categorised, and interpreted as follows:

- 1-3 = not important (item should not be included in extensions)
- 4-6 = important but not critical (item should be discussed)
- 7-9 = important and critical (item should be included in extensions)

Additionally, we will include an “unable to answer” response, for participants who do not feel qualified to rank any specific item [20, 27]. To reduce potential order effect bias ,



For peer review only - http://bmjopen.bmj.com/site/about/guidelines.xhtml

domains (sections with items to rate) will be displayed to study participants in a randomly generated order [43].

In round 1 of the Delphi [20, 40], participants will have the opportunity (through a free text box) to add any proposed modification of items wording and suggestion of additional items [23]. We will consider new items in the second round, if proposed by at least two Delphi participants [44]. Participants can also use the free-text box to explain any of their ratings [23].

Quantitative analysis will be conducted in statistical software such as R [45]. After each round, the proportions for each candidate item will be calculated. In addition, measures to assess the consistency of response (agreement between Delphi rounds) such as median and interquartile range, mean and standard deviations, [27], will be calculated for each item and round. Open text will be analysed using a simple thematic analysis [46] in Microsoft Excel sheets. Results after each round will be shared with the project team before a virtual meeting to discuss modifications and additions needed for the subsequent round [23].

Overall results and participants' own responses in round 1 will be shared with participants in round 2, and they can revise their judgement based on group scoring and explanation for ratings, if any. If conducted, the third round will only include items that have not reached a consensus by round 2 [24, 40]. Delphi studies have no ideal definition of consensus, and definitions vary with contexts [27, 41]. We propose the following consensus definitions based on previous CONSORT extensions [20, 22, 24, 40]:

- Consensus for inclusion: ≥70% participants scoring 7-9 and <15% participants scoring 1-3
- Consensus for exclusion: ≥70% participants scoring 1-3 and <15% of participants scoring 7-9
- No consensus for inclusion or exclusion: failure to achieve both the above

Nevertheless, the project team will have the option to overrule items that achieve a borderline level of consensus (e.g., 65-69% scoring 7-9).

### *Phase 3: Consensus meeting*

The primary objective of the consensus meeting will be to agree on the final items for inclusion in the SPIRIT and CONSORT extensions. Dependent on time availability, this



For peer review only - http://bmjopen.bmj.com/site/about/guidelines.xhtml

meeting will also be used as an opportunity to finalise and refine our knowledge translation strategies for the developed extensions [21]. The meeting will closely follow the EQUATOR Network's guidance on conducting face-to-face consensus meetings [21].

*Structure and participants*

Given the ongoing COVID-19 pandemic and the range of geographical location of participants, we propose to conduct a hybrid meeting with some participants present physically and others joining virtually through an online video conference link. To allow sufficient time to discuss all agenda items [21], and participation from different time zones, we anticipate the meeting will be conducted over two days. Meeting participants will be limited to ≤30 to encourage interaction and debate [21]. Similar to the development of the CONSORT extension for adaptive design [22], we propose the meeting will include the project team members (n=15) and purposively selected stakeholders (n=15). At the end of Delphi survey round 2, participants will be given the opportunity to register their interest in participating in the consensus meeting. The project team will select participants from those interested based on: 1) ability to attend the meeting on proposed dates and times; 2) need to have an international multidisciplinary group of participants.

Prior to the meeting, all participants will be sent (via email): the meeting agenda, participant list, meeting engagement rules, summary of scoping review findings, and Delphi Study results [21]. The meeting will be led by the EC Chair (JSR).

*Consensus procedure*

Items that reached consensus during the Delphi exercise will be discussed and ratified. Items that did not reach consensus will be discussed in detail. The chair will summarise discussions and encourage consensus based on implications (i.e., scientific, ethical, statistical, practical, financial) for inclusion or exclusion of discussed items before voting is conducted. Voting will be conducted anonymously with the following options: "Include in final extension", "Exclude from final extension", "Merge with another item" and "Unsure" [20]. Consensus will be defined as ≥70% voting for either the "include", "exclude" or "merge" options [20, 22, 32]. Items that do not reach consensus will be discussed and a fresh round of voting conducted. This process will continue until consensus is reached or time allocated runs out [20]. If consensus is not reached at the end of the meeting, the PMG and EC will make a final decision [20] at a later virtual meeting held within a month after the consensus meeting. Discussion of items in both meetings will be audio-recorded (with participant verbal consent)



For peer review only - http://bmjopen.bmj.com/site/about/guidelines.xhtml

to help with comprehensive minuting (without direct reference to participants), which will provide a record of decisions taken [21]. Minutes will be shared with all participants after the meeting and archived with other project data.

After discussion of items and dependent on time availability, participants will discuss other agenda items such as the possibility of developing a flow diagram, knowledge translation activities (including authorship of extensions statements), strategies to improve implementation and impact of extensions, and future evaluation of developed outputs [21].

## Phase 4: Knowledge translation

This phase will aim to engage stakeholders and disseminate project outputs, including the developed extensions and will be undertaken throughout the duration of project period.

*Pilot testing and revision of final checklist*

We will pilot the developed SPIRIT and CONSORT extension checklists [21] on a sample of protocols and reports identified from the targeted review. The pilot will involve project team members and other invited researchers and will seek to identify any specific challenges of using the draft checklists and required modifications and inform writing of the explanation and elaboration documents.

*Publications*

We will seek to publish the SPIRIT-SURROGATE and CONSORT-SURROGATE extensions and "Explanation and Elaboration" documents in high impact general medicine journals. To maximise dissemination, we will also seek to co-publish the extensions or editorials/commentaries in other journal settings such as trial-related and public health. We will seek the endorsement of the extensions from journals and editorial groups (e.g., International Committee of Medical Journal Editors) [21]. All project publications will be open access.

*Partner and stakeholder engagement*

The EQUATOR, CONSORT, and SPIRIT groups each provided letters of support for our original funding application for this project and have representatives of EQUATOR (GSC), SPIRIT (AWC) and international SPIRIT/CONSORT-Outcomes Group (NJB, MO) on our advisory Executive Committee. The project is registered on the EQUATOR website [47, 48] and our entry will be regularly updated. We will seek to have the final extensions endorsed and published on the SPIRIT [49] and CONSORT [50] websites. The SPIRIT Statement is



For peer review only - http://bmjopen.bmj.com/site/about/guidelines.xhtml

endorsed by patient groups, trial groups, funders, regulators, and over 100 journals [51], while the CONSORT Statement is endorsed by about 600 journals [52].

We will directly engage potential users of developed extensions, including research funders, healthcare regulators, trial methodologists, and public health and healthcare professionals, to maximise impact. Our project funding application was endorsed by the UK Medical and products Regulatory Agency (MHRA) and the National Institute for Health and Care Excellence (NICE), who will be sent the finalised extensions for implementation. We will seek to reach other stakeholders through presentations at relevant meetings and conferences. We will maintain an active social media presence (i.e., @Consort_surr on Twitter, project team members LinkedIn posts), a project ResearchGate page [53] and project website page (https://www.gla.ac.uk/spirit-consort-surrogate). Finally, we propose to develop video tutorials to illustrate the application of the extensions.

## Patient and public involvement

Patient and public involvement (PPI) will be embedded in all project phases. Our PPI strategy is led by DS who is a member of the PMG and has extensive experience of public involvement in health services research methods. The results of our scoping review will be presented and discussed with PPI representatives in a virtual meeting. We aim to identify ~20 PPI representatives (through snowballing guided by our PPI lead) who will be given introductory training (through a two-hour session) on RCT design and the use of surrogate endpoints to build their capacity to participate in rating items in the Delphi survey. Some of the PPI members who complete the Delphi study will be selected to participate in the consensus meeting based on availability and interest to participate. We will consult our PPI lead on our public/ community dissemination plans. The GRIPP2 (Guidance for Reporting Involvement of Patients and the Public) checklist [54] will be used to guide and report PPI.

## Conclusion

The development of the SPIRIT-SURROGATE and CONSORT-SURROGATE extensions will improve the transparency of reporting and design of RCTs with a surrogate endpoint primary outcome. Such improved reporting should enable the evidence base for surrogate endpoints to be more effectively scrutinised and used for interpretation of trial findings by patients, clinicians and healthcare policy makers.



For peer review only - http://bmjopen.bmj.com/site/about/guidelines.xhtml

## Ethics and dissemination

Ethics approval will be sought from the University of Glasgow College of Medical, Veterinary, and Life Sciences Ethics Committee. Delphi participants will be provided with a participant information sheet and asked to record their online consent. Verbal consent for participation in the consensus meeting (including audio recording of discussions) will be sought at the start of the meeting. Participants will have a right to withdraw from any project activity without giving a reason. We anticipate that all project data collected will not be sensitive and that there is low risk if the identity of participants was exposed. Nevertheless, all data will be securely managed and stored in a participant de-identified form. Delphi participants will be asked to opt-out (via a Yes or No question) if they do not want to be publicly acknowledged in publications. Project outputs will be disseminated through meeting and conference presentations and open-access publications, with stakeholder engagement as outlined in the Phase 4 (knowledge translation) and PPI sections above.

## Contributorship statement

PD, CJW, AY, RT, and OC were involved in funding acquisition. AMM, PD, DS, CJW, AY, RT, and OC were involved in the initial phases of project phases conception and design. AMM, RT, and OC were responsible for the first draft of the manuscript. All authors critically reviewed the first draft and approved the final version. The views expressed in this article are those of the authors and not their employers or funders.

## Competing Interest

Sylwia Bujkiewicz has served as a paid consultant, providing methodological advice, to NICE, Roche and RTI Health Solutions, received payments for educational events from Roche and has received research funding from European Federation of Pharmaceutical Industries & Associations (EEPIA) and Johnson & Johnson. Mario Ouwens works for and has shares in AstraZeneca. Dr Joseph S Ross is an Associate Editor at BMJ and co-founder (unpaid) of medRxiv; Dr. Ross currently receives research support through Yale University from Johnson and Johnson to develop methods of clinical trial data sharing, from the Medical Device Innovation Consortium as part of the National Evaluation System for Health Technology (NEST), from the Food and Drug Administration for the Yale-Mayo Clinic Center for Excellence in Regulatory Science and Innovation (CERSI) program (U01FD005938), from the Agency for Healthcare Research and Quality (R01HS022882),



For peer review only - http://bmjopen.bmj.com/site/about/guidelines.xhtml

from the National Heart, Lung and Blood Institute of the National Institutes of Health (NIH) (R01HS025164, R01HL144644), and from the Laura and John Arnold Foundation to establish the Good Pharma Scorecard at Bioethics International; in addition, Dr. Ross is an expert witness at the request of Relator's attorneys, the Greene Law Firm, in a qui tam suit alleging violations of the False Claims Act and Anti-Kickback Statute against Biogen Inc.

## Funding

The development of SPIRIT and CONSORT extensions has been funded by the UK Medical Research Council (grant number MR/V038400/1). GSC was supported by the NIHR Biomedical Research Centre, Oxford, and Cancer Research UK (programme grant: C49297/A27294).

## References

1. Akobeng, A.K., *Understanding randomised controlled trials.* Archives of Disease in Childhood, 2005. **90**(8): p. 840-844.

2. Hariton, E. and J.J. Locascio, *Randomised controlled trials - the gold standard for effectiveness research: Study design: randomised controlled trials.* BJOG : an international journal of obstetrics and gynaecology, 2018. **125**(13): p. 1716-1716.

3. FDA-NIH Biomarker Working Group, in *BEST (Biomarkers, EndpointS, and other Tools) Resource*. 2021, Food and Drug Administration (US)

National Institutes of Health (US): Silver Spring (MD)

Bethesda (MD).

4. Umscheid, C.A., D.J. Margolis, and C.E. Grossman, *Key concepts of clinical trials: a narrative review.* Postgraduate medicine, 2011. **123**(5): p. 194-204.

5. Biomarkers Definitions Working Group, *Biomarkers and surrogate endpoints: preferred definitions and conceptual framework.* Clin Pharmacol Ther, 2001. **69**(3): p. 89-95.

6. Yetley, E.A., D.L. DeMets, and W.R. Harlan, Jr., *Surrogate disease markers as substitutes for chronic disease outcomes in studies of diet and chronic disease relations.* The American Journal of Clinical Nutrition, 2017. **106**(5): p. 1175-1189.

7. Casarett, D., et al., *Improving the use of hospice services in nursing homes: a randomized controlled trial.* Jama, 2005. **294**(2): p. 211-7.

8. Domke, A., et al., *Immediate effects of a very brief planning intervention on fruit and vegetable consumption: A randomized controlled trial.* Applied Psychology: Health and Well-Being, 2021. **13**(2): p. 377-393.

9. Grimes, D.A. and K.F. Schulz, *Surrogate end points in clinical research: hazardous to your health.* Obstet Gynecol, 2005. **105**(5 Pt 1): p. 1114-8.



For peer review only - http://bmjopen.bmj.com/site/about/guidelines.xhtml

10. Thomas R. Fleming and David L. DeMets, *Surrogate End Points in Clinical Trials: Are We Being Misled?* Annals of Internal Medicine, 1996. **125**(7): p. 605-613.

11. Ciani, O., et al., *Comparison of treatment effect sizes associated with surrogate and final patient relevant outcomes in randomised controlled trials: meta-epidemiological study.* BMJ : British Medical Journal, 2013. **346**: p. f457.

12. Xie, W., et al., *A Systematic Review and Recommendation for Reporting of Surrogate Endpoint Evaluation Using Meta-analyses.* JNCI Cancer Spectr, 2019. **3**(1): p. pkz002.

13. la Cour, J.L., J. Brok, and P.C. Gøtzsche, *Inconsistent reporting of surrogate outcomes in randomised clinical trials: cohort study.* BMJ (Clinical research ed.), 2010. **341**: p. c3653-c3653.

14. Bikdeli, B., et al., *Two Decades of Cardiovascular Trials With Primary Surrogate Endpoints: 1990-2011.* Journal of the American Heart Association, 2017. **6**(3): p. e005285.

15. Chan, A.W., et al., *SPIRIT 2013 statement: defining standard protocol items for clinical trials.* Ann Intern Med, 2013. **158**(3): p. 200-7.

16. Schulz, K.F., D.G. Altman, and D. Moher, *CONSORT 2010 Statement: updated guidelines for reporting parallel group randomised trials.* BMJ, 2010. **340**: p. c332.

17. Turner, L., et al., *Does use of the CONSORT Statement impact the completeness of reporting of randomised controlled trials published in medical journals? A Cochrane review.* Syst Rev, 2012. **1**: p. 60.

18. Calvert, M., et al., *SPIRIT-PRO Extension explanation and elaboration: guidelines for inclusion of patient-reported outcomes in protocols of clinical trials.* BMJ Open, 2021. **11**(6): p. e045105.

19. Calvert, M., et al., *Reporting of Patient-Reported Outcomes in Randomized Trials: The CONSORT PRO Extension.* JAMA, 2013. **309**(8): p. 814-822.

20. Butcher, N.J., et al., *Improving outcome reporting in clinical trial reports and protocols: study protocol for the Instrument for reporting Planned Endpoints in Clinical Trials (InsPECT).* Trials, 2019. **20**(1): p. 161.

21. Moher, D., et al., *Guidance for developers of health research reporting guidelines.* PLoS Med, 2010. **7**(2): p. e1000217.

22. Dimairo, M., et al., *Development process of a consensus-driven CONSORT extension for randomised trials using an adaptive design.* BMC Medicine, 2018. **16**(1): p. 210.

23. Imran, M., et al., *Methods and results used in the development of a consensus-driven extension to the Consolidated Standards of Reporting Trials (CONSORT) statement for trials conducted using cohorts and routinely collected data (CONSORT-ROUTINE).* BMJ Open, 2021. **11**(4): p. e049093.

24. Kwakkenbos, L., et al., *Protocol for the development of a CONSORT extension for RCTs using cohorts and routinely collected health data.* Research Integrity and Peer Review, 2018. **3**(1): p. 9.

25. Manyara, A.M., Davies, P., Stewart, D., Wells, V., Weir, C. J., Young, A., Taylor, R., Ciani, O.,. *Protocol for Scoping and Targeted Reviews to Support Development of SPIRIT*

For peer review only



For peer review only - http://bmjopen.bmj.com/site/about/guidelines.xhtml

*and CONSORT Extensions for Randomised Controlled Trials with Surrogate Primary Endpoints*. 2022 03 March 2022].

26. Arksey, H. and L. O'Malley, *Scoping studies: towards a methodological framework.* International Journal of Social Research Methodology, 2005. **8**(1): p. 19-32.

27. Trevelyan, E.G. and P.N. Robinson, *Delphi methodology in health research: how to do it?* European Journal of Integrative Medicine, 2015. **7**(4): p. 423-428.

28. Goodman, C.M., *The Delphi technique: a critique.* Journal of Advanced Nursing, 1987. **12**(6): p. 729-734.

29. Hsu, C.-C. and B.A. Sandford, *The Delphi technique: making sense of consensus.* Practical assessment, research, and evaluation, 2007. **12**(1): p. 10.

30. Murphy, M., et al., *Consensus development methods, and their use in clinical guideline development.* Health Technology Assessment (Winchester, England), 1998. **2**(3): p. i-88.

31. Liu, X., et al., *Reporting guidelines for clinical trial reports for interventions involving artificial intelligence: the CONSORT-AI extension.* Nature Medicine, 2020. **26**(9): p. 1364-1374.

32. Duarte, R.V., et al., *Reporting Guidelines for Clinical Trial Protocols and Reports of Implantable Neurostimulation Devices: Protocol for the SPIRIT-iNeurostim and CONSORT-iNeurostim Extensions.* Neuromodulation, 2021.

33. Thabane, L., et al., *Methods and processes for development of a CONSORT extension for reporting pilot randomized controlled trials.* Pilot and Feasibility Studies, 2016. **2**(1): p. 25.

34. Rivera, S.C., et al., *Guidelines for clinical trial protocols for interventions involving artificial intelligence: the SPIRIT-AI Extension.* BMJ, 2020. **370**: p. m3210.

35. Montgomery, P., et al., *Reporting randomised trials of social and psychological interventions: the CONSORT-SPI 2018 Extension.* Trials, 2018. **19**(1): p. 407.

36. Hopewell, S., et al., *CONSORT for reporting randomized controlled trials in journal and conference abstracts: explanation and elaboration.* PLoS medicine, 2008. **5**(1): p. e20.

37. Black, N., et al., *Consensus Development Methods: A Review of Best Practice in Creating Clinical Guidelines.* Journal of Health Services Research & Policy, 1999. **4**(4): p. 236-248.

38. Ritchie, J., J. Lewis, and G. Elam, *Designing and selecting samples.* Qualitative research methods, 2003: p. 77-108.

39. Christina Yap, M.A.E., et al., *Development of a CONSORT Extension for Early-Phase DoseFinding Trials (DF-CONSORT)*. 2021.

40. Montgomery Alan , et al., *Reporting of randomised factorial trials: development of extensions to the CONSORT 2010 and SPIRIT 2013 guidance statements*. 2021.

41. Keeney, S., F. Hasson, and H. McKenna, *Consulting the oracle: ten lessons from using the Delphi technique in nursing research.* Journal of Advanced Nursing, 2006. **53**(2): p. 205-212.



For peer review only - http://bmjopen.bmj.com/site/about/guidelines.xhtml

42. Guyatt, G.H., et al., *GRADE guidelines: 2. Framing the question and deciding on important outcomes.* J Clin Epidemiol, 2011. **64**(4): p. 395-400.

43. Berger, J., *Does Presentation Order Impact Choice After Delay?* Topics in Cognitive Science, 2016. **8**(3): p. 670-684.

44. de Blacam, C., et al., *Protocol for the development of a core outcome set for reporting outcomes of management of velopharyngeal dysfunction.* BMJ Open, 2020. **10**(8): p. e036824.

45. R Core Team, *R: A language and environment for statistical computing*. 2013, Vienna, Austria.

46. Braun, V. and V. Clarke, *Using thematic analysis in psychology.* Qualitative research in psychology, 2006. **3**(2): p. 77-101.

47. EQUATOR Network. *CONSORT-SURROGATE – CONSORT extension for trials using surrogate primary endpoints (registered 10 March 2022)*. 2022; Available from: https://www.equator-network.org/library/reporting-guidelines-under-development/reporting-guidelines-under-development-for-clinical-trials/#SURROGATE.

48. EQUATOR Network. *SPIRIT-SURROGATE – SPIRIT extension for protocols of studies using surrogate primary endpoints (registered 10 March 2022)*. 2022; Available from: https://www.equator-network.org/library/reporting-guidelines-under-development/reporting-guidelines-under-development-for-clinical-trials-protocols/#SURROGATE.

49. SPIRIT Statement. *SPIRIT Statement Website*. 2022; Available from: https://www.spirit-statement.org/.

50. CONSORT Statement. *CONSORT Statement Website*. 2022; Available from: http://www.consort-statement.org/.

51. SPIRIT Statement. *Endorsement*. 2022 02/03/2022]; Available from: https://www.spirit-statement.org/about-spirit/spirit-endorsement/.

52. CONSORT Statement. *Endorsers*. 2022 02/03/2022]; Available from: http://www.consort-statement.org/about-consort/endorsers1.

53. ResearchGate. *Development of surrogate endpoint reporting guidelines: SPIRIT-SURROGATE and CONSORT-SURROGATE*. 2022 2 March 2022]; Available from: https://www.researchgate.net/project/Development-of-surrogate-endpoint-reporting-guidelines-SPIRIT-SURROGATE-and-CONSORT-SURROGATE.

54. Staniszewska, S., et al., *GRIPP2 reporting checklists: tools to improve reporting of patient and public involvement in research.* BMJ, 2017. **358**: p. j3453.

## Acknowledgments

The authors would like to thank Valerie Wells (University of Glasgow) for helping with development of a search strategy for the literature reviews and Rui Duarte and Rebecca Bresnahan (both from University of Liverpool) for their advice during conceptualisation of Delphi study design.



For peer review only - http://bmjopen.bmj.com/site/about/guidelines.xhtml

# Figure legend

Figure 1: Project phases, timelines, activities in each phase (middle), with integrated knowledge translation (left) and patient and public involvement (right). Timelines include preparatory work before start of each phase. Adapted from [24].

RCTs – Randomised Controlled Trials

For peer review only



For peer review only - http://bmjopen.bmj.com/site/about/guidelines.xhtml

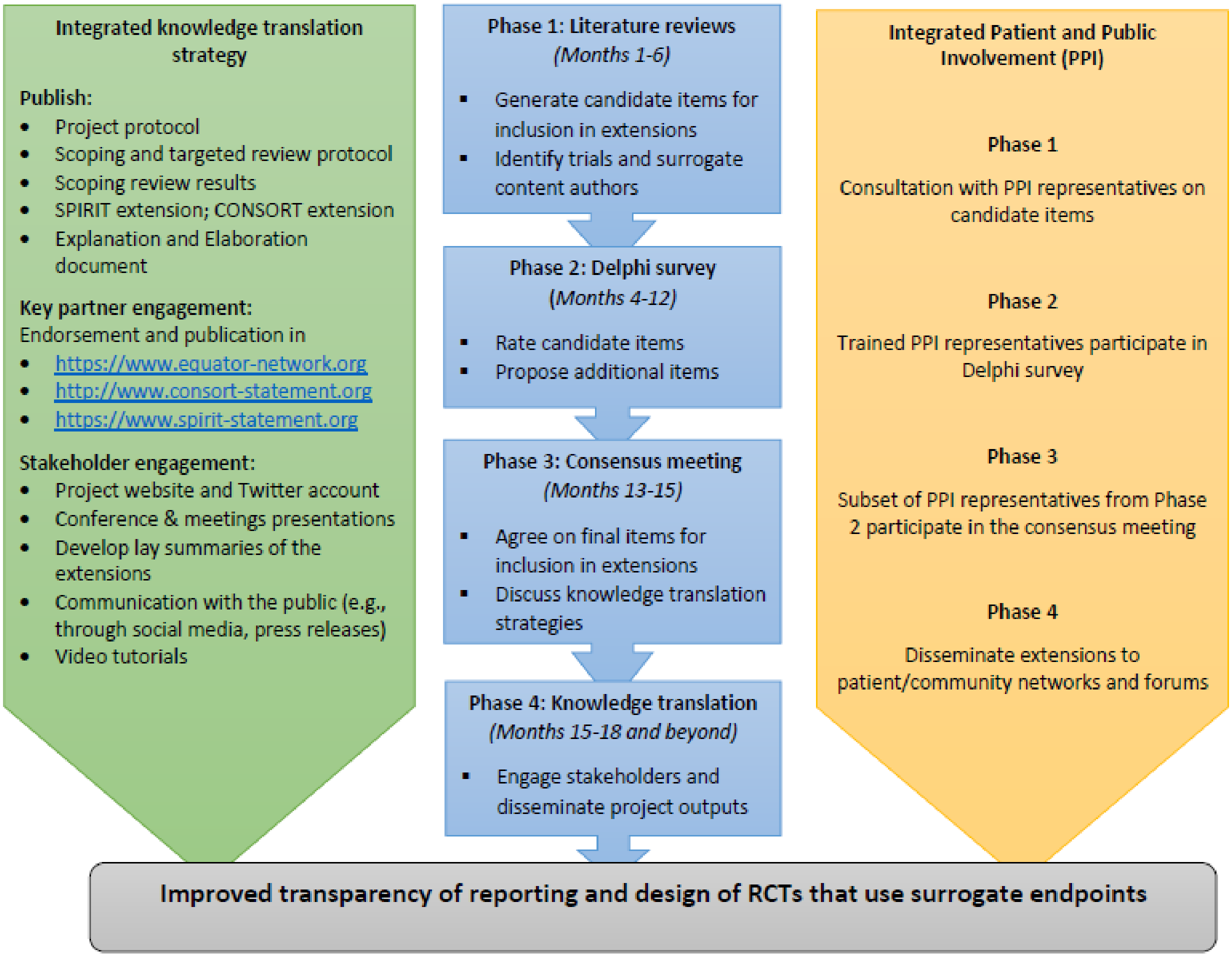
Integrated knowledge translation strategy
Publish:
Project protocol
Scoping and targeted review protocol
Scoping review results
SPIRIT extension; CONSORT extension
Explanation and Elaboration document
Key partner engagement:
Endorsement and publication in
https://www.equator-network.org
http://www.consort-statement.org
https://www.spirit-statement.org
Stakeholder engagement:
Project website and Twitter account
Conference & meetings presentations
Develop lay summaries of the extensions
Communication with the public (e.g., through social media, press releases)
Video tutorials
Phase 1: Literature reviews
(Months 1-6)
Generate candidate items for inclusion in extensions
Identify trials and surrogate content authors
Phase 2: Delphi survey
(Months 4-12)
Rate candidate items
Propose additional items
Phase 3: Consensus meeting
(Months 13-15)
Agree on final items for inclusion in extensions
Discuss knowledge translation strategies
Phase 4: Knowledge translation
(Months 15-18 and beyond)
Engage stakeholders and disseminate project outputs
Integrated Patient and Public Involvement (PPI)
Phase 1
Consultation with PPI representatives on candidate items
Phase 2
Trained PPI representatives participate in Delphi survey
Phase 3
Subset of PPI representatives from Phase 2 participate in the consensus meeting
Phase 4
Disseminate extensions to patient/community networks and forums
Improved transparency of reporting and design of RCTs that use surrogate endpoints

1

# Supplementary File

**Supplementary Table 1: Delphi survey stakeholder categories (approximate target sample sizes, and identification strategies)**

| Stakeholder category | Approx. sample: % (n) | Identification strategies |
|---|---|---|
| **Methodologists, Healthcare professionals, and Epidemiologists based in academia and healthcare industry** | ~50% (100) | • Professional bodies and networks such as the UKCRC CTU network<br>• Conferences and meetings such as the 6th International Clinical Trials Methodology Conference 2022 (ictmc.org/)<br>• Corresponding authors of protocols and trials identified in the Targeted Review (Phase 1)<br>• Projects team members professional contacts |
| **Surrogate content experts** | ~20% (40) | • Corresponding authors of literature included in the scoping review (Phase 1)<br>• Project team members professional contacts |
| **Public and patient representatives** | ~10% (20) | • Patient and public engagement forums such as European Patient Forum (www.eu-patient.eu)<br>• Consumers/patient and public involvement experts in the MRC-NIHR Trials Methodology Research Partnership<br>• Project team members professional contacts |
| **Funders, regulators, HTA experts and clinical guideline developers** | ~10% (20) | • Funding committees' members (with special interest in clinical trials) such as the NIHR/MRC<br>• Health technology assessment bodies such as NICE<br>• Regulatory bodies such as MHRA, FDA<br>• Project team members professional contacts |
| **Journal editors** | ~10% (20) | • Editorial groups such as the International Committee of Medical Journal Editors<br>• Project team professional contacts |
| **A call will be placed on the project website and social media pages and targeted invitations for potential participants from any stakeholder category to register**<br><br>***Weighting will not be done during analysis** | | |

For peer review only

BMJ Open

# Protocol for the development of SPIRIT and CONSORT extensions for randomised controlled trials with surrogate primary endpoints: SPIRIT-SURROGATE and CONSORT-SURROGATE.

| | |
|---|---|
| Journal: | *BMJ Open* |
| Manuscript ID | bmjopen-2022-064304.R1 |
| Article Type: | Protocol |
| Date Submitted by the Author: | 08-Aug-2022 |
| Complete List of Authors: | Manyara, Anthony; University of Glasgow<br>Davies, Philippa ; University of Bristol<br>Stewart, Derek; Patient and Public Involvement lead<br>Weir, Christopher; University of Edinburgh, Edinburgh Clinical Trials Unit, Usher Institute<br>Young, Amber; University Hospitals Bristol NHS Foundation Trust, Paediatric Anaesthesia; University of Bristol, Canynge Hall<br>Butcher, Nancy; Sick Kids, Child Health Evaluative Sciences<br>Bujkiewicz, Sylwia; Biostatistics Research Group, Department of Health Sciences, University of Leicester<br>Chan, An-Wen; University of Toronto, Medicine<br>Collins, Gary; Oxford University, Centre for Statistics in Medicine<br>Dawoud, Dalia; National Institute for Health and Care Excellence<br>Offringa, Martin; The Hospital for Sick Children, Child Health Evaluation Sciences<br>Ouwens, Mario; AstraZeneca<br>Ross, Joseph; Yale University School of Medicine, Internal Medicine<br>Taylor, Rod; University of Glasgow<br>Ciani, Oriana ; Università Bocconi |
| <b>Primary Subject Heading</b>: | Research methods |
| Secondary Subject Heading: | Medical publishing and peer review |
| Keywords: | STATISTICS & RESEARCH METHODS, Protocols & guidelines < HEALTH SERVICES ADMINISTRATION & MANAGEMENT, GENERAL MEDICINE (see Internal Medicine), PUBLIC HEALTH |
| | |

SCHOLARONE™
Manuscripts

# Protocol for the development of SPIRIT and CONSORT extensions for randomised controlled trials with surrogate primary endpoints: SPIRIT-SURROGATE and CONSORT-SURROGATE.

Anthony Muchai Manyara[1], Philippa Davies[2], Derek Stewart[3], Christopher J Weir[4], Amber E Young[2], Nancy J. Butcher[5,6], Sylwia Bujkiewicz[7]. An-Wen Chan[8,9], Gary S Collins[10], Dalia Dawoud[11], Martin Offringa[5], Mario Ouwens[12], Joseph S. Ross[13,14], Rod S Taylor[1,15], Oriana Ciani[16]

1 MRC/CSO Social and Public Health Sciences Unit, Institute of Health and Wellbeing, University of Glasgow, Glasgow, UK

2 Population Health Sciences, Bristol Medical School, University of Bristol, Bristol, UK

3 Patient and Public Involvement lead, UK

4 Edinburgh Clinical Trials Unit, Usher Institute, University of Edinburgh, Edinburgh, UK

5 Child Health Evaluative Sciences, The Hospital for Sick Children Research Institute, Toronto, Canada

6 Department of Psychiatry, University of Toronto, Toronto, Canada

7 Biostatistics Research Group, Department of Health Sciences, University of Leicester, Leicester, UK

8 Women's College Institute Research Institute, Toronto, Canada

9 Department of Medicine, University of Toronto, Toronto, Canada

10 Centre for Statistics in Medicine, Nuffield Department of Orthopaedics, Rheumatology & Musculoskeletal Sciences, University of Oxford, Oxford, UK

11 Science, Evidence and Analytics Directorate, Science Policy and Research Programme, National Institute for Health and Care Excellence, London, UK

12 AstraZeneca, Weesp, Netherlands

13 Department of Health Policy and Management, Yale School of Public Health, New Haven, CT, USA



For peer review only - http://bmjopen.bmj.com/site/about/guidelines.xhtml

14 Section of General Medicine, Department of Internal Medicine, Yale School of Medicine, New Haven, CT, USA

15 Robertson Centre for Biostatistics, Institute of Health and Well Being, University of Glasgow, Glasgow, UK

16 SDA Bocconi School of Management, Milan, Italy

Correspondence to Anthony Manyara; Anthony.Manyara@glasgow.ac.uk

MRC/CSO Social and Public Health Sciences Unit, Institute of Health and Wellbeing, University of Glasgow, Glasgow, UK

For peer review only



For peer review only - http://bmjopen.bmj.com/site/about/guidelines.xhtml

# Abstract

*Introduction*

Randomised controlled trials (RCTs) may use surrogate endpoints as substitutes and predictors of patient/participant-relevant final outcomes (e.g., survival, health related quality of life). Translation of effects measured on a surrogate endpoint into health benefits for patients/participants is dependent on the validity of the surrogate, hence more accurate and transparent reporting on surrogate endpoints is needed to limit misleading interpretation of trial findings. However, there is currently no explicit guidance for the reporting of such trials. Therefore, we aim to develop extensions to the SPIRIT (Standard Protocol Items: Recommendations for Interventional Trials) and CONSORT (Consolidated Standards of Reporting Trials) reporting guidelines to improve the design and completeness of reporting of RCTs and their protocols using a surrogate endpoint as a primary outcome.

*Methods and analysis*

The project will have four phases: Phase 1 (literature reviews) to identify candidate reporting items to be rated in a Delphi study; Phase 2 (Delphi study) to rate the importance of items identified in Phase 1 and receive suggestions for additional items; Phase 3 (consensus meeting) to agree on final set of items for inclusion in the extensions; and Phase 4 (knowledge translation) to engage stakeholders and disseminate the project outputs through various strategies including peer-reviewed publications. Patient and public involvement will be embedded into all project phases.

*Ethics and dissemination*

The study has received ethical approval from the University of Glasgow College of Medical, Veterinary, and Life Sciences Ethics Committee (Project No: 200210051). The findings will be published in open-access peer-reviewed publications and presented in conferences, meetings, and relevant forums.


For peer review only



For peer review only - http://bmjopen.bmj.com/site/about/guidelines.xhtml

## Strengths and limitations of this study

- We will follow the EQUATOR Network's recommended steps for developing a health research reporting guideline.
- The Delphi study will target an international and multidisciplinary group of participants.
- Patient and public involvement will be integrated in all phases of the study.
- Although we will target an international participation, Delphi study and consensus meeting will be conducted in English which could limit participation from non-English speaking settings.

For peer review only



For peer review only - http://bmjopen.bmj.com/site/about/guidelines.xhtml

# Introduction

Evidence for the effectiveness of interventions should ideally come from randomised controlled trials (RCTs) [1 2] that assess a patient /study participant relevant final outcome (PRFO): a measurement that reflects how an individual feels, functions, or survives [3] such as mortality or health-related quality of life. Nevertheless, in order to meet the scientific, ethical, and regulatory requirements, the conduct and delivery of many RCTs has become increasingly resource and time-intensive [4]. By reducing follow up time, sample size, and cost, a surrogate endpoint that substitutes for a PRFO, can provide a potentially attractive solution for improving trial efficiency[5]. This efficiency allows for early detection of intervention effects[6] which could lead to accelerated approval of interventions prior to confirmation of benefit on the PRFO[7] or when there is lack of effect, stopping of trials or roll out of interventions with no health benefit. .

Over the last decade, a number of biomarkers [an objectively measured molecular, histologic, radiographic, or physiologic characteristic used as an indicator of response to an intervention[5]] have been accepted as surrogate endpoints in the regulatory approval of pharmaceuticals and medical devices e.g., blood pressure and low density lipoprotein-cholesterol within the cardiovascular disease context [8]. However, for a wider range of interventions including surgical, organizational, and public health interventions, the so-called intermediate outcomes [i.e., an outcome that can be measured earlier than an effect on PRFO and is predictive of the intervention effect on the PRFO] [3]] have been used with the aim of replacing and predicting for a PRFO (e.g., hospice enrolment instead of mortality with an intervention aimed at improving end of life care [9]; fruit and vegetable consumption instead of cardiovascular events for a behavioural intervention designed to improve cardiovascular risk [10]). To be accepted as a valid surrogate endpoint, a biomarker or intermediate outcome needs to both: 1) reliably predict the PRFO in individual participants ('individual level' or 'patient-level' surrogacy); and 2) the intervention effect on surrogate endpoint should reliably predict the intervention effect on the PRFO ('trial-level' surrogacy) which is assessed through a meta-analytic model of data on both outcomes typically obtained from RCTs[11 12]. In summary, surrogacy validation depends on the use of various statistical methods, including meta-analysis of trial aggregate data and/or individual patient data[13 14], principal stratification [15], causal inference [16 17], information theory [18], and bivariate network meta-analysis methods[19 20].



For peer review only - http://bmjopen.bmj.com/site/about/guidelines.xhtml

Despite their potential benefits, the use of surrogate endpoints in health and policy decision making remains controversial. Some regulatory approvals based on a surrogate endpoint have led to the use of interventions that have resulted in more harm than health benefit for patients due to the surrogate not being in the pathway of the PRFO or unintended effects of the intervention [21 22 23 24]. Additionally, RCTs using a surrogate endpoint primary outcome have been shown to overestimate treatment effects (adjusted odds ratios: 1.46, 95% CI: 1.05 to 2.04) compared with RCTs with PRFO as a primary outcome [25]. It is therefore important that RCTs using surrogate endpoints are appropriately designed and reported e.g., explicitly state that the primary outcome is a surrogate endpoint, give a clear rationale for its use, and provide evidence of its validation (i.e., prior evidence of the association between the intervention effect on the putative surrogate endpoint and PRFO) [7]. However, a retrospective analysis of 626 RCTs published in 2005 and 2006 found that 109 (17%) used a surrogate primary endpoint and of these, only a third discussed whether the surrogate endpoint was a valid predictor of health benefit on a PRFO [26]. Similarly, a more recent review of 220 cardiovascular intervention trials using surrogate biomarkers found that only 59 (27%) had confirmatory evidence validating the benefits of interventions on a PRFO [27].

Reporting guidelines can improve transparency and completeness in the reporting of RCTs at both the protocol and final report stages. The two prominent trial guidelines are: SPIRIT (Standard Protocol Items: Recommendations for Interventional Trials) 2013 statement: a 33-item checklist used to report RCT protocols [28]; and CONSORT (Consolidated Standards of Reporting Trials) 2010 statement is a 25-item checklist, which has improved reporting of completed trials [29 30]. Although SPIRIT and CONSORT and related extensions, including the (SPIRIT-PRO [31], CONSORT-PRO [32]) and the ongoing CONSORT-Outcomes [33], provide general guidance on trial outcome reporting, there remains no standard evidence-based guideline for the reporting of RCTs with a surrogate primary outcome. Therefore, in this study, we aim to develop specific extensions to report RCT protocols and final trial reports that use a surrogate primary endpoint: SPIRIT-SURROGATE and CONSORT-SURROGATE, respectively. These extensions will improve the transparency of reporting and design of RCTs with a surrogate endpoint as a primary outcome. Such improved reporting should enable the evidence base for surrogate endpoints to be more effectively scrutinised and used for interpretation of trial findings by patients, clinicians, and healthcare policy makers. This protocol describes the methods that will be used in developing these extensions.



For peer review only - http://bmjopen.bmj.com/site/about/guidelines.xhtml

# Methods

Our methodology will be guided by: the EQUATOR (Enhancing the QUAlity and Transparency Of health Research) Network's recommended steps for developing a health research reporting guideline [34]; and methodological considerations used to develop other recent or ongoing SPIRIT/CONSORT extensions (e.g., ACE [Adaptive designs CONSORT Extension] [35], CONSORT-ROUTINE [36], Dose-Finding CONSORT Extension [37]).

The project will be overseen by a Project Management Group (PMG) and an advisory Executive Committee (EC). The PMG includes the lead investigators (OC, RST) and project manager (AMM) responsible for the day-to-day management of the project plus project co-investigators (PD, AEY, CJW), and patient and public involvement lead (DS). The EC is an international and multidisciplinary group (JSR [chair], NJB, SB, AWC, GSC, DD, MO, MO) providing strategic oversight of the project and will contribute to the dissemination, endorsement, and implementation of developed extensions.

Figure 1 shows the project phases, timelines, activities in each phase, and the integrated knowledge translation and patient and public involvement which are described in more detail below.

## *Phase 1: Literature reviews*

A detailed protocol for this phase has been prepared, prospectively registered [38], and submitted for publication. Briefly, we will undertake two separate literature reviews that include a scoping review and a 'targeted review'. The scoping review, conducted using the Arksey and O'Malley six-stage methodological framework [39], will seek to explore *the current understanding, limitations, acceptability, and guidance on using surrogate endpoints in RCTs* and generate two outputs: candidate items ("long-list") for rating through a Delphi exercise; and a contact list of surrogate content experts who will be invited to participate in the exercise. Further, one of the issues to be explored in the scoping review will be an appropriate and comprehensive working definition of surrogate endpoint, starting from the most commonly reported definition in 2001 by the National Institutes of Health (i.e., a biomarker or laboratory measure intended to substitute and predict for a variable that reflects how patients feel, function or how long they survive) [5]. The targeted review will seek to systematically identify recent protocols and full reports of RCTs that have used surrogate endpoints as primary outcomes. It will serve two purposes: 1) identify researchers who have used surrogate endpoints who will be invited to participate in the Delphi exercise; and 2) the



For peer review only - http://bmjopen.bmj.com/site/about/guidelines.xhtml

identified protocols and reports will inform a detailed contemporary analysis of the completeness of reporting of RCTs with surrogate endpoint primary outcomes.

### *Phase 2: Delphi study*

The primary objective of this phase will be to rate candidate items generated in Phase 1. Secondary objectives will be to identify additional items not included in the initial list; and allow for modifications in the wording of important items [36].

*Study design and setting*

The Delphi methodology is a widely used consensus-building technique whose main features are: use of experts as participants, anonymity between participants, iterations and controlled feedback (to allow for "communication" and consensus building between participants), and summary of participant views [40-43]. Its key practical advantage is the non-requirement of face-to-face contact [43], enabling more participants and broader representation in terms of geography and key groups in the development of health research reporting guidelines [34]. Whilst our virtual Delphi approach provides participant anonymity that may allow for more open expression of views [41], it has also the potential disadvantage of lack of group interaction which can contribute to consensus building [43]. However, the final consensus meeting (see below) will have an in-person element.

Our Delphi study will be conducted online and facilitated by DelphiManager software (version 5.0), a bespoke software developed and maintained by the COMET (Core Outcome Measures in Effectiveness Trials) initiative (https://www.comet-initiative.org/), which has been used to develop other SPIRIT and CONSORT extensions [44 45].

It is recommended that the number of Delphi study rounds is determined *a priori* [40]. To meet project timelines and reduce potential participant burden/fatigue, we propose two rounds will be conducted. Two Delphi rounds have been used to develop SPIRIT/CONSORT extensions for reporting: pilot RCTs [46]; RCTs using adaptive design [35]; interventions involving artificial intelligence [44 47]; and social and psychological interventions [48]. However, if we fail to obtain consensus in most items after round 2 and based on advice from the EC, we will consider a third round [33 36 49].

*Sample size, recruitment, and inclusion criteria*

Delphi studies do not require formal sample size calculations, although the number and characteristics of participants need to be carefully considered [40 42 43 50]. Consistent with the EQUATOR Network's guidance [34], we will seek to recruit an international multidisciplinary



For peer review only - http://bmjopen.bmj.com/site/about/guidelines.xhtml

group of stakeholders, including trialists, trial methodologists, statisticians, healthcare professionals, researchers, content experts, journal editors, patient and public representatives, funders, regulators, Health Technology Assessment experts and clinical guideline developers. Supplementary File 1 shows stakeholder categories, approximate target sample sizes, and strategies for access. We will target to have at least 200 participants register interest to take part in the study. A sample size of ≥200 will allow recruitment of at least 20 participants per stakeholder group and reasonable numbers (>70 participants) to complete the study, assuming ≥60% response rate of eligible participants and ≤40% attrition (in the context of completing all Delphi rounds), both as observed in previous Delphi studies that have developed extension guidelines [35 36 46 47].

We will use purposive and snowball sampling (nonprobability sampling) to include participants [51]. Identification strategies will include: 1) professional contacts known to the PMG and EC; 2) relevant professional bodies and networks; 3) relevant conferences and meetings; 4) authors of records included in the scoping and targeted reviews (Phase 1); 5) a call for participants on project website and social media pages; 6) asking registered participants to share link with other people, networks or organisations that would be interested in participating (see Supplementary table in Supplementary File); and 7) we have published short articles [52 53 54] to create awareness of the project and signpost readers to the project website to register interest in participation . We acknowledge that our target sample size may be challenging, particularly for some stakeholder categories (e.g., journal editors (n=20)); however, efforts will be made to reach as many of these stakeholders as possible.

Participant inclusion criteria will be: 1) expertise in surrogate endpoints (through authored literature) or self-reported interest and basic understanding of the concept of surrogacy; 2) registered interest, in English (nevertheless participation will be international), to participate during the allocated period. We will have no exclusion criteria. During registration, the following descriptive data on participants will be collected: self-identified stakeholder group (primary/secondary roles), clinical or research area (if actively involved), country of work, and self-reported basic understanding of surrogate endpoints.

*Data collection, analysis, and consensus definition*

Prior to the launch of the first survey round, a pilot (with n=15 participants) will be conducted to gauge user-friendliness, improve wording/logical flow, and identify any practical concerns [55]. Following the pilot, all registered participants will be emailed a web



For peer review only - http://bmjopen.bmj.com/site/about/guidelines.xhtml

link prompting them to complete any of the Delphi rounds. The weblink will access the Delphi survey landing page that includes: a short text section emphasising the importance of completing the exercise [43], a participant information sheet, and a consent checkbox. Each round will be open for approximately four weeks, and the second round sent out 3-4 weeks after the closure of the first. Email reminders to complete the survey will be sent to participants to improve response rates. Participants who complete all Delphi rounds may opt to enter a prize draw to win one of two £100 vouchers [56].

Candidate Delphi items will be ranked using a Likert scale. There is no consensus on the ideal rating scale in Delphi studies [57]. We will use the Grading of Recommendations Assessment, Development and Evaluations (GRADE) 9-point scale [58], that has been used in other extensions [33 35 44-46 56], categorised, and interpreted as follows:

- 1-3 = not important (item should not be included in extensions)
- 4-6 = important but not critical (item should be discussed)
- 7-9 = important and critical (item should be included in extensions)

Additionally, we will include an "unable to answer" response, for participants who do not feel qualified to rank any specific item [33 40]. In round 1 of the Delphi [33 56], participants will have the opportunity (through a free text box) to add any proposed modification of items wording and suggestion of additional items [36]. We will consider new items in the second round, if proposed by at least two Delphi participants [59]. Participants can also use the free-text box to explain any of their ratings [36].

Quantitative analysis will be conducted in statistical software such as R [60]. After each round, the proportions for each candidate item will be calculated. In addition, measures to assess the consistency of response (agreement between Delphi rounds) such as median and interquartile range, mean and standard deviations, [40], will be calculated for each item and round. Open text will be analysed using a simple thematic analysis [61] in Microsoft Excel sheets. Results after each round will be shared with the project team before a virtual meeting to discuss modifications and additions needed for the subsequent round [36].

Overall results and participants' own responses in round 1 will be shared with participants in round 2, and they can revise their judgement based on group scoring and explanation for ratings, if any. If conducted, the third round will only include items that have not reached a consensus by round 2 [56 62]. Delphi studies have no ideal definition of consensus, and



For peer review only - http://bmjopen.bmj.com/site/about/guidelines.xhtml

definitions vary with contexts [40,57]. We propose the following consensus definitions based on previous CONSORT extensions [33,35,56,62]:

- Consensus for inclusion: ≥70% participants scoring 7-9 and <15% participants scoring 1-3
- Consensus for exclusion: ≥70% participants scoring 1-3 and <15% of participants scoring 7-9
- No consensus for inclusion or exclusion: failure to achieve both the above

Nevertheless, the project team will have the option to overrule items that achieve a borderline level of consensus (e.g., 65-69% scoring 7-9).

***Phase 3: Consensus meeting***

The primary objective of the consensus meeting will be to agree on the final items for inclusion in the SPIRIT and CONSORT extensions. Dependent on time availability, this meeting will also be used as an opportunity to finalise and refine our knowledge translation strategies for the developed extensions [34]. The meeting will closely follow the EQUATOR Network's guidance on conducting face-to-face consensus meetings [34].

*Structure and participants*

Given the ongoing COVID-19 pandemic and the range of geographical location of participants, we propose to conduct a hybrid meeting with some participants present physically and others joining virtually through an online video conference link. To allow sufficient time to discuss all agenda items [34], and participation from different time zones, we anticipate the meeting will be conducted over two days. Meeting participants will be limited to ≤30 to encourage interaction and debate [34]. Similar to the development of the CONSORT extension for adaptive design [35], we propose the meeting will include the project team members (n=15) and purposively selected stakeholders (n=15). At the end of Delphi survey round 2, participants will be given the opportunity to register their interest in participating in the consensus meeting. The project team will select participants from those interested based on: 1) ability to attend the meeting on proposed dates and times; 2) need to have an international multidisciplinary group of participants.

Prior to the meeting, all participants will be sent (via email): the meeting agenda, participant list, meeting engagement rules, summary of scoping review findings, and Delphi Study results [34]. The meeting will be led by the EC Chair (JSR).



For peer review only - http://bmjopen.bmj.com/site/about/guidelines.xhtml

*Consensus procedure*

Items that reached consensus during the Delphi exercise will be discussed and ratified. Items that did not reach consensus will be discussed in detail. The chair will summarise discussions and encourage consensus based on implications (i.e., scientific, ethical, statistical, practical, financial) for inclusion or exclusion of discussed items before voting is conducted. Voting will be conducted anonymously with the following options: "Include in final extension", "Exclude from final extension", "Merge with another item" and "Unsure" [33]. Consensus will be defined as ≥70% voting for either the "include", "exclude" or "merge" options [33 35 45]. Items that do not reach consensus will be discussed and a fresh round of voting conducted. This process will continue until consensus is reached or time allocated runs out [33]. If consensus is not reached at the end of the meeting, the PMG and EC will make a final decision [33] at a later virtual meeting held within a month after the consensus meeting. Discussion of items in both meetings will be audio-recorded (with participant verbal consent) to help with comprehensive minuting (without direct reference to participants), which will provide a record of decisions taken [34]. Minutes will be shared with all participants after the meeting and archived with other project data.

After discussion of items and dependent on time availability, participants will discuss other agenda items such as the possibility of developing a flow diagram, knowledge translation activities (including authorship of extensions statements), strategies to improve implementation and impact of extensions, and future evaluation of developed outputs [34].

## Phase 4: Knowledge translation

This phase will aim to engage stakeholders and disseminate project outputs, including the developed extensions and will be undertaken throughout the duration of project period.

*Pilot testing and revision of final checklist*

We will pilot the developed SPIRIT and CONSORT extension checklists [34] on a sample of protocols and reports identified from the targeted review. The pilot will involve project team members and other invited researchers and will seek to identify any specific challenges of using the draft checklists and required modifications and inform writing of the explanation and elaboration documents.

*Publications*

We will seek to publish the SPIRIT-SURROGATE and CONSORT-SURROGATE extensions and "Explanation and Elaboration" documents in high impact general medicine



For peer review only - http://bmjopen.bmj.com/site/about/guidelines.xhtml

journals. To maximise dissemination, we will also seek to co-publish the extensions or editorials/commentaries in other journal settings such as trial-related and public health. We will seek the endorsement of the extensions from journals and editorial groups (e.g., International Committee of Medical Journal Editors) [34]. All project publications will be open access.

*Partner and stakeholder engagement*

The EQUATOR, CONSORT, and SPIRIT groups each provided letters of support for our original funding application for this project and have representatives of EQUATOR (GSC), SPIRIT (AWC) and international SPIRIT/CONSORT-Outcomes Group (NJB, MO) on our advisory Executive Committee. The project is registered on the EQUATOR website [63 64] and our entry will be regularly updated. We will seek to have the final extensions endorsed and published on the SPIRIT [65] and CONSORT [66] websites. The SPIRIT Statement is endorsed by patient groups, trial groups, funders, regulators, and over 100 journals [67], while the CONSORT Statement is endorsed by about 600 journals [68].

We will directly engage potential users of developed extensions, including research funders, healthcare regulators, trial methodologists, and public health and healthcare professionals, to maximise impact. Our project funding application was endorsed by the UK Medical and products Regulatory Agency (MHRA) and the National Institute for Health and Care Excellence (NICE), who will be sent the finalised extensions for implementation. We will seek to reach other stakeholders through presentations at relevant meetings and conferences. We will maintain an active social media presence (i.e., @Consort_surr on Twitter, project team members LinkedIn posts), a project ResearchGate page [69] and project website page (https://www.gla.ac.uk/spirit-consort-surrogate). Finally, we propose to develop video tutorials to illustrate the application of the extensions.

## Patient and public involvement

Patient and public involvement (PPI) will be embedded in all project phases. Our PPI strategy is led by DS who is a member of the PMG and has extensive experience of public involvement in health services research methods. The results of our scoping review will be presented and discussed with PPI representatives in a virtual meeting. We aim to identify ~20 PPI representatives (through snowballing guided by our PPI lead) who will be given introductory training (through a two-hour session) on RCT design and the use of surrogate endpoints to build their capacity to participate in rating items in the Delphi survey. Some of



For peer review only - http://bmjopen.bmj.com/site/about/guidelines.xhtml

the PPI members who complete the Delphi study will be selected to participate in the consensus meeting based on availability and interest to participate. We will consult our PPI lead on our public/ community dissemination plans. The GRIPP2 (Guidance for Reporting Involvement of Patients and the Public) checklist [70] will be used to guide and report PPI.

## Ethics and dissemination

The study has received ethical approval from the University of Glasgow College of Medical, Veterinary, and Life Sciences Ethics Committee (Project No: 200210051). Delphi participants will be provided with a participant information sheet and asked to record their online consent. Verbal consent for participation in the consensus meeting (including audio recording of discussions) will be sought at the start of the meeting. Participants will have a right to withdraw from any project activity without giving a reason. We anticipate that all project data collected will not be sensitive and that there is low risk if the identity of participants was exposed. Nevertheless, all data will be securely managed and stored in a participant de-identified form. Delphi participants will be asked to opt-out (via a Yes or No question) if they do not want to be publicly acknowledged in publications. Project outputs will be disseminated through meeting and conference presentations and open-access publications, with stakeholder engagement as outlined in the Phase 4 (knowledge translation) and PPI sections above.

## Contributorship statement

PD, CJW, AY, RT, and OC were involved in funding acquisition. AMM, PD, DS, CJW, AY, RT, and OC were involved in the initial phases of project phases conception and design. AMM, RT, and OC were responsible for the first draft of the manuscript. NJB, SB, AWC, GSC, DD, MO, MO, and JSR critically reviewed the first draft and approved the final version. The views expressed in this article are those of the authors and not their employers or funders.

## Competing Interests

Sylwia Bujkiewicz has served as a paid consultant, providing methodological advice, to NICE, Roche and RTI Health Solutions, received payments for educational events from Roche and has received research funding from European Federation of Pharmaceutical Industries & Associations (EEPIA) and Johnson & Johnson. Mario Ouwens works for and has shares in AstraZeneca. Dr Joseph S Ross is an Associate Editor at BMJ and co-founder



For peer review only - http://bmjopen.bmj.com/site/about/guidelines.xhtml

(unpaid) of medRxiv; Dr. Ross currently receives research support through Yale University from Johnson and Johnson to develop methods of clinical trial data sharing, from the Medical Device Innovation Consortium as part of the National Evaluation System for Health Technology (NEST), from the Food and Drug Administration for the Yale-Mayo Clinic Center for Excellence in Regulatory Science and Innovation (CERSI) program (U01FD005938), from the Agency for Healthcare Research and Quality (R01HS022882), from the National Heart, Lung and Blood Institute of the National Institutes of Health (NIH) (R01HS025164, R01HL144644), and from the Laura and John Arnold Foundation to establish the Good Pharma Scorecard at Bioethics International; in addition, Dr. Ross is an expert witness at the request of Relator's attorneys, the Greene Law Firm, in a qui tam suit alleging violations of the False Claims Act and Anti-Kickback Statute against Biogen Inc.

## Funding

The development of SPIRIT and CONSORT extensions has been funded by the UK Medical Research Council (grant number MR/V038400/1). GSC was supported by the NIHR Biomedical Research Centre, Oxford, and Cancer Research UK (programme grant: C49297/A27294).

## References

1. Akobeng AK. Understanding randomised controlled trials. *Archives of Disease in Childhood* 2005;90(8):840-44. doi: 10.1136/adc.2004.058222

2. Hariton E, Locascio JJ. Randomised controlled trials - the gold standard for effectiveness research: Study design: randomised controlled trials. *BJOG* 2018;125(13):1716-16. doi: 10.1111/1471-0528.15199 [published Online First: 2018/06/19]

3. FDA-NIH Biomarker Working Group. BEST (Biomarkers, EndpointS, and other Tools) Resource. Silver Spring (MD)

Bethesda (MD): Food and Drug Administration (US)

National Institutes of Health (US) 2021.

4. Umscheid CA, Margolis DJ, Grossman CE. Key concepts of clinical trials: a narrative review. *Postgraduate medicine* 2011;123(5):194-204. doi: 10.3810/pgm.2011.09.2475

5. Biomarkers Definitions Working Group. Biomarkers and surrogate endpoints: preferred definitions and conceptual framework. *Clin Pharmacol Ther* 2001;69(3):89-95. doi: 10.1067/mcp.2001.113989 [published Online First: 2001/03/10]

6. Fleming TR, Powers JH. Biomarkers and surrogate endpoints in clinical trials. *Statistics in Medicine* 2012;31(25):2973-84. doi: https://doi.org/10.1002/sim.5403



For peer review only - http://bmjopen.bmj.com/site/about/guidelines.xhtml

For peer review only

7. FDA-NIH Biomarker Working Group. BEST (Biomarkers, endpoints, and other tools) resource [Internet]. 2021

8. Yetley EA, DeMets DL, Harlan WR, Jr. Surrogate disease markers as substitutes for chronic disease outcomes in studies of diet and chronic disease relations. *The American Journal of Clinical Nutrition* 2017;106(5):1175-89. doi: 10.3945/ajcn.117.164046

9. Casarett D, Karlawish J, Morales K, et al. Improving the use of hospice services in nursing homes: a randomized controlled trial. *Jama* 2005;294(2):211-7. doi: 10.1001/jama.294.2.211 [published Online First: 2005/07/15]

10. Domke A, Keller J, Heuse S, et al. Immediate effects of a very brief planning intervention on fruit and vegetable consumption: A randomized controlled trial. *Applied Psychology: Health and Well-Being* 2021;13(2):377-93. doi: https://doi.org/10.1111/aphw.12254

11. Buyse M, Molenberghs G, Burzykowski T, et al. The validation of surrogate endpoints in meta-analyses of randomized experiments. *Biostatistics* 2000;1(1):49-67. doi: 10.1093/biostatistics/1.1.49 [published Online First: 2003/08/23]

12. Buyse M, Saad ED, Burzykowski T, et al. Surrogacy Beyond Prognosis: The Importance of "Trial-Level" Surrogacy. *The Oncologist* 2022;27(4):266-71. doi: 10.1093/oncolo/oyac006

13. Xie W, Halabi S, Tierney JF, et al. A Systematic Review and Recommendation for Reporting of Surrogate Endpoint Evaluation Using Meta-analyses. *JNCI Cancer Spectr* 2019;3(1):pkz002. doi: 10.1093/jncics/pkz002 [published Online First: 2019/07/31]

14. Daniels MJ, Hughes MD. Meta-analysis for the evaluation of potential surrogate markers. *Stat Med* 1997;16(17):1965-82. doi: 10.1002/(sici)1097-0258(19970915)16:17<1965::aid-sim630>3.0.co;2-m [published Online First: 1997/09/26]

15. Weir CJ, Walley RJ. Statistical evaluation of biomarkers as surrogate endpoints: a literature review. *Stat Med* 2006;25(2):183-203. doi: 10.1002/sim.2319 [published Online First: 2005/10/28]

16. Alonso A, Van der Elst W, Molenberghs G, et al. On the relationship between the causal-inference and meta-analytic paradigms for the validation of surrogate endpoints. *Biometrics* 2015;71(1):15-24. doi: 10.1111/biom.12245 [published Online First: 2014/10/03]

17. Vanderweele TJ. Surrogate measures and consistent surrogates. *Biometrics* 2013;69(3):561-9. doi: 10.1111/biom.12071 [published Online First: 2013/10/01]

18. Alonso A, Van der Elst W, Molenberghs G, et al. An information-theoretic approach for the evaluation of surrogate endpoints based on causal inference. *Biometrics* 2016;72(3):669-77. doi: 10.1111/biom.12483 [published Online First: 2016/02/13]

19. Bujkiewicz S, Jackson D, Thompson JR, et al. Bivariate network meta-analysis for surrogate endpoint evaluation. *Stat Med* 2019;38(18):3322-41. doi: 10.1002/sim.8187 [published Online First: 2019/05/28]

20. Papanikos T, Thompson JR, Abrams KR, et al. Bayesian hierarchical meta-analytic methods for modeling surrogate relationships that vary across treatment classes using aggregate data. *Stat Med* 2020;39(8):1103-24. doi: 10.1002/sim.8465 [published Online First: 2020/01/29]



For peer review only - http://bmjopen.bmj.com/site/about/guidelines.xhtml

21. Grimes DA, Schulz KF. Surrogate end points in clinical research: hazardous to your health. *Obstet Gynecol* 2005;105(5 Pt 1):1114-8. doi: 10.1097/01.aog.0000157445.67309.19 [published Online First: 2005/05/03]

22. Thomas R. Fleming, David L. DeMets. Surrogate End Points in Clinical Trials: Are We Being Misled? *Annals of Internal Medicine* 1996;125(7):605-13. doi: 10.7326/0003-4819-125-7-199610010-00011 %m 8815760

23. Cohen D. Rosiglitazone: what went wrong? *Bmj* 2010;341:c4848. doi: 10.1136/bmj.c4848 [published Online First: 2010/09/08]

24. Kumar S, Rajkumar SV. Surrogate endpoints in randomised controlled trials: a reality check. *Lancet* 2019;394(10195):281-83. doi: 10.1016/s0140-6736(19)31711-8 [published Online First: 2019/07/30]

25. Ciani O, Buyse M, Garside R, et al. Comparison of treatment effect sizes associated with surrogate and final patient relevant outcomes in randomised controlled trials: meta-epidemiological study. *BMJ : British Medical Journal* 2013;346:f457. doi: 10.1136/bmj.f457

26. la Cour JL, Brok J, Gøtzsche PC. Inconsistent reporting of surrogate outcomes in randomised clinical trials: cohort study. *BMJ* 2010;341:c3653-c53. doi: 10.1136/bmj.c3653

27. Bikdeli B, Punnanithinont N, Akram Y, et al. Two Decades of Cardiovascular Trials With Primary Surrogate Endpoints: 1990-2011. *J Am Heart Assoc* 2017;6(3):e005285. doi: 10.1161/JAHA.116.005285

28. Chan AW, Tetzlaff JM, Altman DG, et al. SPIRIT 2013 statement: defining standard protocol items for clinical trials. *Ann Intern Med* 2013;158(3):200-7. doi: 10.7326/0003-4819-158-3-201302050-00583 [published Online First: 2013/01/09]

29. Schulz KF, Altman DG, Moher D. CONSORT 2010 Statement: updated guidelines for reporting parallel group randomised trials. *BMJ* 2010;340:c332. doi: 10.1136/bmj.c332

30. Turner L, Shamseer L, Altman DG, et al. Does use of the CONSORT Statement impact the completeness of reporting of randomised controlled trials published in medical journals? A Cochrane review. *Syst Rev* 2012;1:60. doi: 10.1186/2046-4053-1-60 [published Online First: 2012/12/01]

31. Calvert M, King M, Mercieca-Bebber R, et al. SPIRIT-PRO Extension explanation and elaboration: guidelines for inclusion of patient-reported outcomes in protocols of clinical trials. *BMJ Open* 2021;11(6):e045105. doi: 10.1136/bmjopen-2020-045105

32. Calvert M, Blazeby J, Altman DG, et al. Reporting of Patient-Reported Outcomes in Randomized Trials: The CONSORT PRO Extension. *JAMA* 2013;309(8):814-22. doi: 10.1001/jama.2013.879

33. Butcher NJ, Monsour A, Mew EJ, et al. Improving outcome reporting in clinical trial reports and protocols: study protocol for the Instrument for reporting Planned Endpoints in Clinical Trials (InsPECT). *Trials* 2019;20(1):161. doi: 10.1186/s13063-019-3248-0

34. Moher D, Schulz KF, Simera I, et al. Guidance for developers of health research reporting guidelines. *PLoS medicine* 2010;7(2):e1000217. doi: 10.1371/journal.pmed.1000217 [published Online First: 2010/02/20]



For peer review only - http://bmjopen.bmj.com/site/about/guidelines.xhtml

35. Dimairo M, Coates E, Pallmann P, et al. Development process of a consensus-driven CONSORT extension for randomised trials using an adaptive design. *BMC Medicine* 2018;16(1):210. doi: 10.1186/s12916-018-1196-2

36. Imran M, Kwakkenbos L, McCall SJ, et al. Methods and results used in the development of a consensus-driven extension to the Consolidated Standards of Reporting Trials (CONSORT) statement for trials conducted using cohorts and routinely collected data (CONSORT-ROUTINE). *BMJ Open* 2021;11(4):e049093. doi: 10.1136/bmjopen-2021-049093

37. Yap C, Bedding A, de Bono J, et al. The need for reporting guidelines for early phase dose-finding trials: Dose-Finding CONSORT Extension. *Nature Medicine* 2022;28(1):6-7. doi: 10.1038/s41591-021-01594-1

38. Manyara AM, Davies, P., Stewart, D., Wells, V., Weir, C. J., Young, A., Taylor, R., Ciani, O.,. Protocol for Scoping and Targeted Reviews to Support Development of SPIRIT and CONSORT Extensions for Randomised Controlled Trials with Surrogate Primary Endpoints: OSF; 2022 [accessed 03 March 2022.

39. Arksey H, O'Malley L. Scoping studies: towards a methodological framework. *International Journal of Social Research Methodology* 2005;8(1):19-32. doi: 10.1080/1364557032000119616

40. Trevelyan EG, Robinson PN. Delphi methodology in health research: how to do it? *European Journal of Integrative Medicine* 2015;7(4):423-28. doi: https://doi.org/10.1016/j.eujim.2015.07.002

41. Goodman CM. The Delphi technique: a critique. *Journal of Advanced Nursing* 1987;12(6):729-34. doi: https://doi.org/10.1111/j.1365-2648.1987.tb01376.x

42. Hsu C-C, Sandford BA. The Delphi technique: making sense of consensus. *Practical assessment, research, and evaluation* 2007;12(1):10.

43. Murphy M, Black N, Lamping D, et al. Consensus development methods, and their use in clinical guideline development. *Health Technology Assessment (Winchester, England)* 1998;2(3):i-88.

44. Liu X, Cruz Rivera S, Moher D, et al. Reporting guidelines for clinical trial reports for interventions involving artificial intelligence: the CONSORT-AI extension. *Nature Medicine* 2020;26(9):1364-74. doi: 10.1038/s41591-020-1034-x

45. Duarte RV, Bresnahan R, Copley S, et al. Reporting Guidelines for Clinical Trial Protocols and Reports of Implantable Neurostimulation Devices: Protocol for the SPIRIT-iNeurostim and CONSORT-iNeurostim Extensions. *Neuromodulation* 2021 doi: 10.1016/j.neurom.2021.10.006 [published Online First: 2022/01/29]

46. Thabane L, Hopewell S, Lancaster GA, et al. Methods and processes for development of a CONSORT extension for reporting pilot randomized controlled trials. *Pilot and Feasibility Studies* 2016;2(1):25. doi: 10.1186/s40814-016-0065-z

47. Rivera SC, Liu X, Chan A-W, et al. Guidelines for clinical trial protocols for interventions involving artificial intelligence: the SPIRIT-AI Extension. *BMJ* 2020;370:m3210. doi: 10.1136/bmj.m3210



For peer review only - http://bmjopen.bmj.com/site/about/guidelines.xhtml

48. Montgomery P, Grant S, Mayo-Wilson E, et al. Reporting randomised trials of social and psychological interventions: the CONSORT-SPI 2018 Extension. *Trials* 2018;19(1):407. doi: 10.1186/s13063-018-2733-1

49. Hopewell S, Clarke M, Moher D, et al. CONSORT for reporting randomized controlled trials in journal and conference abstracts: explanation and elaboration. *PLoS medicine* 2008;5(1):e20.

50. Black N, Murphy M, Lamping D, et al. Consensus Development Methods: A Review of Best Practice in Creating Clinical Guidelines. *Journal of Health Services Research & Policy* 1999;4(4):236-48. doi: 10.1177/135581969900400410

51. Ritchie J, Lewis J, Elam G. Designing and selecting samples. *Qualitative research methods* 2003:77-108.

52. Ciani O, Manyara AM, Taylor RS. Surrogate endpoints in trials—a call for better reporting. *BMJ* 2022;378:o1912. doi: 10.1136/bmj.o1912

53. Manyara AM, Ciani O, Taylor RS. A call for better reporting of trials using surrogate primary endpoints. *Alzheimer's & Dementia: Translational Research & Clinical Interventions* 2022;8(1):e12340. doi: https://doi.org/10.1002/trc2.12340

54. Ciani O, Manyara A, Taylor RS. Need for better reporting of trials with surrogate endpoints: SPIRIT|CONSORT-SURROGATE extensions. *Journal of Epidemiology and Community Health* 2022:jech-2022-219294. doi: 10.1136/jech-2022-219294

55. Christina Yap MAE, Olga Solovyeva, Adrian Mander, et al. Development of a CONSORT Extension for Early-Phase DoseFinding Trials (DF-CONSORT), 2021.

56. Montgomery Alan , Kahan Brennan, Elbourne Diana , et al. Reporting of randomised factorial trials: development of extensions to the CONSORT 2010 and SPIRIT 2013 guidance statements, 2021.

57. Keeney S, Hasson F, McKenna H. Consulting the oracle: ten lessons from using the Delphi technique in nursing research. *Journal of Advanced Nursing* 2006;53(2):205-12. doi: https://doi.org/10.1111/j.1365-2648.2006.03716.x

58. Guyatt GH, Oxman AD, Kunz R, et al. GRADE guidelines: 2. Framing the question and deciding on important outcomes. *J Clin Epidemiol* 2011;64(4):395-400. doi: 10.1016/j.jclinepi.2010.09.012 [published Online First: 2011/01/05]

59. de Blacam C, Baylis AL, Kirschner RE, et al. Protocol for the development of a core outcome set for reporting outcomes of management of velopharyngeal dysfunction. *BMJ Open* 2020;10(8):e036824. doi: 10.1136/bmjopen-2020-036824

60. R Core Team. R: A language and environment for statistical computing: Vienna, Austria, 2013.

61. Braun V, Clarke V. Using thematic analysis in psychology. *Qualitative research in psychology* 2006;3(2):77-101.

62. Kwakkenbos L, Juszczak E, Hemkens LG, et al. Protocol for the development of a CONSORT extension for RCTs using cohorts and routinely collected health data. *Research Integrity and Peer Review* 2018;3(1):9. doi: 10.1186/s41073-018-0053-3



For peer review only - http://bmjopen.bmj.com/site/about/guidelines.xhtml

63. EQUATOR Network. CONSORT-SURROGATE – CONSORT extension for trials using surrogate primary endpoints (registered 10 March 2022) 2022 [22/03/2022,]. Available from: https://www.equator-network.org/library/reporting-guidelines-under-development/reporting-guidelines-under-development-for-clinical-trials/#SURROGATE.

64. EQUATOR Network. SPIRIT-SURROGATE – SPIRIT extension for protocols of studies using surrogate primary endpoints (registered 10 March 2022) 2022 [22/03/2022,]. Available from: https://www.equator-network.org/library/reporting-guidelines-under-development/reporting-guidelines-under-development-for-clinical-trials-protocols/#SURROGATE.

65. SPIRIT Statement. SPIRIT Statement Website 2022 [02/03/2022]. Available from: https://www.spirit-statement.org/.

66. CONSORT Statement. CONSORT Statement Website 2022 [02/03/2022]. Available from: http://www.consort-statement.org/.

67. SPIRIT Statement. Endorsement 2022 [Available from: https://www.spirit-statement.org/about-spirit/spirit-endorsement/ accessed 02/03/2022.

68. CONSORT Statement. Endorsers 2022 [Available from: http://www.consort-statement.org/about-consort/endorsers1 accessed 02/03/2022.

69. ResearchGate. Development of surrogate endpoint reporting guidelines: SPIRIT-SURROGATE and CONSORT-SURROGATE 2022 [02/03/2022]. Available from: https://www.researchgate.net/project/Development-of-surrogate-endpoint-reporting-guidelines-SPIRIT-SURROGATE-and-CONSORT-SURROGATE accessed 2 March 2022.

70. Staniszewska S, Brett J, Simera I, et al. GRIPP2 reporting checklists: tools to improve reporting of patient and public involvement in research. *BMJ* 2017;358:j3453. doi: 10.1136/bmj.j3453

## Acknowledgments

The authors would like to thank Valerie Wells (University of Glasgow) for helping with development of a search strategy for the literature reviews and Rui Duarte and Rebecca Bresnahan (both from University of Liverpool) for their advice during conceptualisation of Delphi study design.

## Figure legend

Figure 1: Project phases, timelines, activities in each phase (middle), with integrated knowledge translation (left) and patient and public involvement (right). Timelines include preparatory work before start of each phase. Adapted from [62].

RCTs – Randomised Controlled Trials



For peer review only - http://bmjopen.bmj.com/site/about/guidelines.xhtml

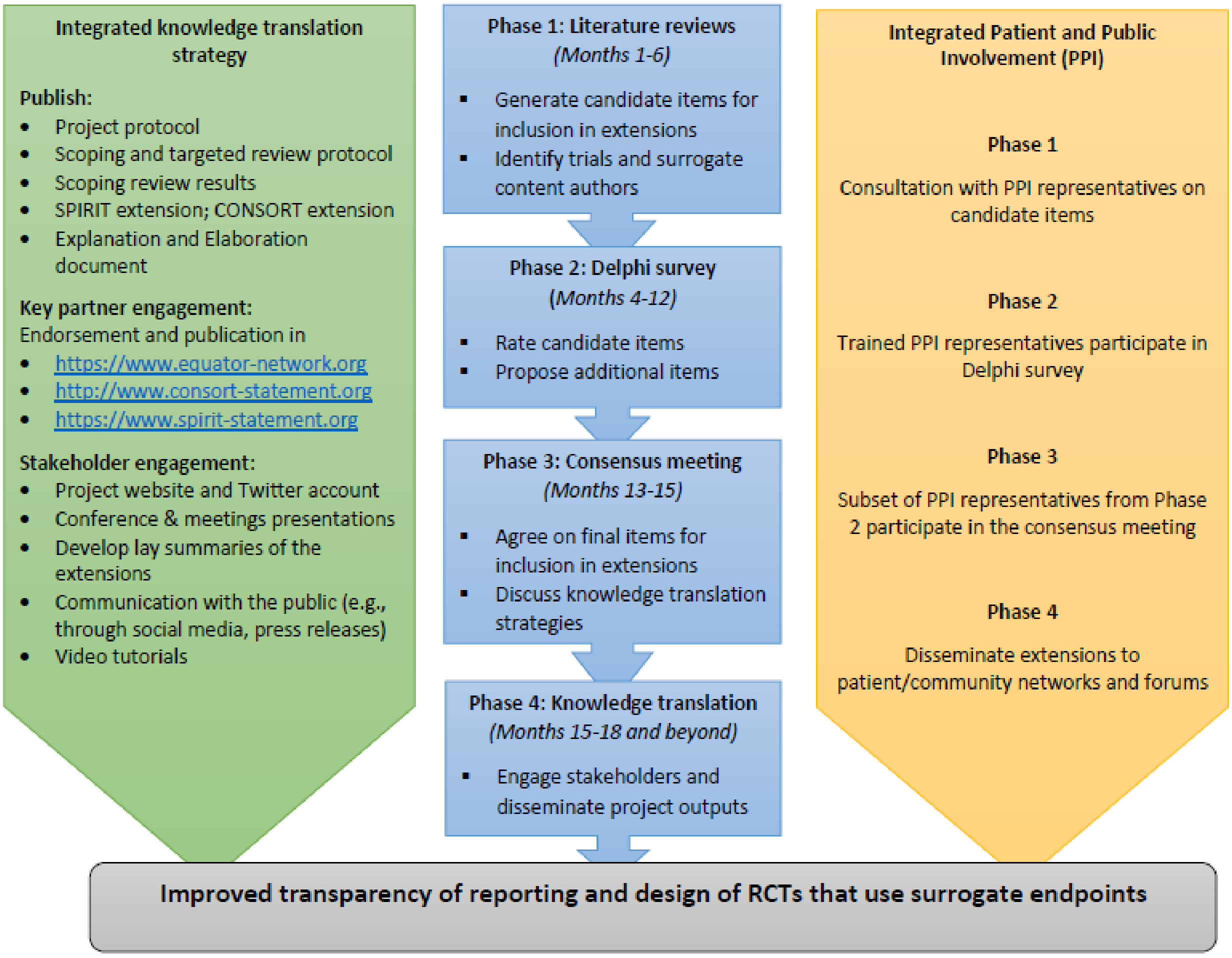


ew only



For peer review only - http://bmjopen.bmj.com/site/about/guidelines.xhtml

# Supplementary File

**Supplementary Table 1: Delphi survey stakeholder categories (approximate target sample sizes, and identification strategies)**

| Stakeholder category | Approx. sample: % (n) | Identification strategies |
|---|---|---|
| **Methodologists, Statisticians, Healthcare professionals, and Epidemiologists based in academia and healthcare industry** | ~50% (100) | • Professional bodies and networks such as the UKCRC CTU network<br>• Conferences and meetings such as the 6th International Clinical Trials Methodology Conference 2022 (ictmc.org/)<br>• Corresponding authors of protocols and trials identified in the Targeted Review (Phase 1)<br>• Projects team members professional contacts |
| **Surrogate content experts** | ~20% (40) | • Corresponding authors of literature included in the scoping review (Phase 1)<br>• Project team members professional contacts |
| **Public and patient representatives** | ~10% (20) | • Patient and public engagement forums such as European Patient Forum (www.eu-patient.eu)<br>• Consumers/patient and public involvement experts in the MRC-NIHR Trials Methodology Research Partnership<br>• Project team members professional contacts |
| **Funders, regulators, HTA experts and clinical guideline developers** | ~10% (20) | • Funding committees' members (with special interest in clinical trials) such as the NIHR/MRC<br>• Health technology assessment bodies such as NICE<br>• Regulatory bodies such as MHRA, FDA<br>• Project team members professional contacts |
| **Journal editors** | ~10% (20) | • Editorial groups such as the International Committee of Medical Journal Editors<br>• Project team professional contacts |

**A call will be placed on the project website and social media pages and targeted invitations for potential participants from any stakeholder category to register**

***Weighting will not be done during analysis**

For peer review only

For peer review only
